# TRAF2 decreases lipid accumulation in hepatocytes under endoplasmic reticulum stress: TRAF2 increases the ubiquitination of XBP1s

**DOI:** 10.3724/abbs.2023094

**Published:** 2023-07-04

**Authors:** Siqi Li, Yang Li, Xiaoxia Wang, Zhixiong Xia, Ronggui Hu

**Affiliations:** 1 School of Medicine Guizhou University Guiyang 550025 China; 2 College of Life Science and Technology Key Laboratory of Molecular Biophysics of MOE and International Research Center for Sensory Biology and Technology of MOST Huazhong University of Science and Technology Wuhan 430074 China; 3 Shanghai Institute of Immunology Department of Immunology and Microbiology Shanghai Jiao Tong University School of Medicine Shanghai Jiao Tong University Shanghai 200025 China; 4 Key Laboratory of Systems Health Science of Zhejiang Province School of Life Science Hangzhou Institute for Advance Study University of Chinese Academy of Sciences Hangzhou 310024 China; 5 State Key Laboratory of Molecular Biology Shanghai Institute of Biochemistry and Cell Biology Center for Excellence in Molecular Cell Science Chinese Academy of Sciences Shanghai 200031 China

The liver plays a pivotal role in the regulation of lipid and glucose metabolism. However, increased intake of nutrients, including glucose, fructose, and saturated fat, induces hepatic *de novo* lipogenesis and subclinical inflammation in the adipose tissue and liver [1]. Deregulated nutrients perturb endoplasmic reticulum (ER) homeostasis and activate the stress response pathway unfolding protein response (UPR). There are three well-known signaling pathways in the UPR: inositol-requiring enzyme 1α/X-box binding protein 1 (IRE1α/XBP1), protein kinase R-like endoplasmic reticulum kinase/eukaryotic initiation factor 2α (PERK/eIF2α), and activating transcription factor 6 (ATF6).

The most conserved signaling pathway in the UPR is IRE1α/XBP1s. Numerous studies have suggested that IRE1α/XBP1s regulates nutrient metabolism in the liver. The role of IRE1α/XBP1s in lipid metabolism has been established using many mammalian models [ 2, 3]. Moreover, IRE1α/XBP1s reportedly plays a similar role in *Caenorhabditis elegans*. Chronic exposure to environmental stress disrupts lipid metabolism in worms, and the role of IRE-1 and HSP-4 in energy homeostasis in *C*. *elegans* has been reported [4]. Therefore, there may be a conserved pathway for ER stress-related lipid metabolism in worms and mammalian tissues.

TNF receptor-associated factor 2 (TRAF2) is associated with NF-κB activation. The TRAF family consists of seven members in the human genome. All TRAF proteins contain an N-terminal RING domain that exists in E3 ligases and several zinc finger domains; however, TRAF1 only contains the N-terminal RING domain. Among the TRAF proteins, TRAF2 and TRAF6 have been most extensively studied [5]. *C*. *elegans* contains two analog proteins, *trf-1* and *trf-2*. According to sequencing analysis, *trf-1* is the gene that is most analogous to *TRAF2* in humans and *Traf2* in mice. Previous studies have demonstrated that tunicamycin (TM)- or thapsigargin (TG)-induced ER stress increases the degradation of TRAF2 in the L929 cell line [6]. Generally, the IRE1α pathway has three arms: IRE1α splices *XBP1* to *XBP1s*, regulates IRE1α-dependent decay using the RNase domain, and recruits TRAF2 by IRE1α and initiates the JNK/NF-κB pathway. *Traf2* and *Xbp1s* play crucial roles in metabolic responses in mouse models [ 7, 8]. However, the crosstalk between IRE1α/XBP1s and IRE1α/TRAF2 remains unclear.

To demonstrate the role of TRAF2 in lipid metabolism, a *TRAF2*-expressing plasmid was transfected into the normal hepatocyte Lo2 cell line. Treatment with palmitic acid (PA), a saturated fatty acid, remarkably increased lipid (labelled with BODIPY 505/515) accumulation that could be completely inhibited by the overexpression of TRAF2 ( Figure 1A). Consistent with previous studies, we found that PA treatment activated the IRE1α/XBP1s and PERK/eIF2α pathways, indicating ER stress. Phosphorylated IRE1α and eIF2α were increased with PA treatment. The protein level of XBP1s was significantly increased under PA treatment ( Figure 1B). Moreover, *TRAF2* knockdown in Lo2 cells ( Figure 1C) substantially increased lipid droplets ( Figure 1D). These findings imply that TRAF2 negatively regulates lipid metabolism.

**Figure 1:**
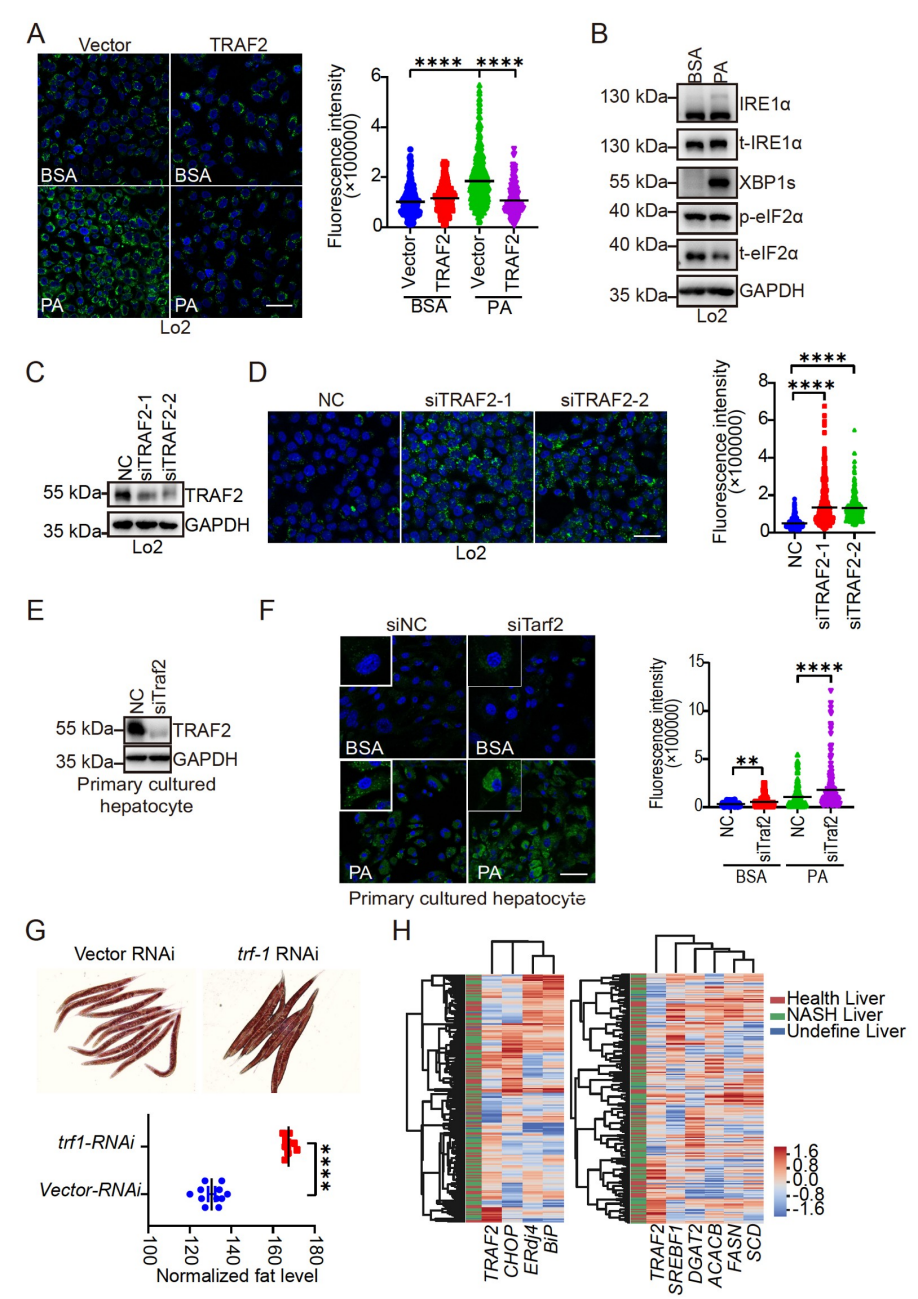
Figure 1 A conserved role of TRAF2 in lipid metabolism from *H*. *sapiens* to *C*. *elegans* (A) The Lo2 cell line was transfected with vector or TRAF2-expressing plasmid for 24 h and subjected to treatment with 10% BSA or palmitic acid (0.5 mM PA in 10% BSA) for 24 h. Cells were stained with BODIPY 505/515 (green) and DAPI (blue). The green dots (lipid droplets) were quantified per cell, and the quantification results are presented on the right of the panel. (B) Lo2 cells were treated with 10% BSA or PA (0.5 mM) for 24 h. Cell lysate was analyzed by western blot analysis with indicated antibodies. (C,D) Lo2 cells were transfected with indicated RNA oligos for 72 h. (C) TRAF2 expression was analyzed by western blot analysis and (D) transfected cells were subjected to BODIPY (green) and DAPI (blue) staining. (E,F) Primary cultured hepatocytes were isolated from wild-type C57BJ mice. Cells were transfected with indicated siRNA of Traf2 (siTraf2) for 72 h and TRAF2 expression was analyzed (E). (F) Representative images (left) and quantitative analysis (right) of lipid droplet staining. (G) Adult worms were fed with indicated RNAi bacteria from eggs. Oil Red O staining illustrates lipid accumulation in C. elegans (upper panel); quantitative analysis of lipid accumulation (lower panel). (H) The expression patterns of indicated genes in the GSE83452 dataset. Scale bar: 50 μM. Data are presented as the mean±SEM from three independent experiments. **P<0.01, and ****P<0.0001 (two-tailed unpaired Student’s t -test).

IRE1α/XBP1s and TRAF2 are conserved in many species, including humans, mice, and *C*. *elegans*. To demonstrate that the regulation of lipid accumulation is conserved in other species, we knocked down *Traf2* in primary cultured hepatocytes isolated from C57BL mice ( Figure 1E). We noticed a significant accumulation of lipid droplets in primary cultured hepatocytes with *Traf2* knockdown after PA treatment ( Figure 1F). Furthermore, worms were fed with *trf-1* RNAi bacteria to knock down *trf-1*. Oil Red O staining indicated that *trf-1* knockdown in worms increased lipid accumulation ( Figure 1G). We further analyzed the human liver with or without nonalcoholic steatohepatitis hepatitis (NASH) using a GEO dataset [9] and found a negative correlation between the mRNA level of *TRAF2* and ER stress signaling [including the mRNA levels of *CHOP* ( *DDIT3*), *ERdj4* ( *DNAJB9*), and *BiP* ( *HSPA5*)] and lipid metabolism signaling (including the mRNA levels of *SREBF1*, *DGAT2*, *ACACB*, *FASN*, and *SCD*) ( Figure 1H). These findings indicate the conservation of the role of TRAF2 in lipid metabolism from *Homo sapiens* to *C*. *elegans*.

TRAF2 is a RING domain E3 ligase. We overexpressed TRAF2 and XBP1s in HEK293T cells to further investigate the crosstalk between TRAF2 and IRE1α/XBP1s signaling. Our results revealed that TRAF2 dramatically increased the ubiquitination of XBP1s ( Figure 2A). XBP1s is a transcription factor that targets many stress-responsive genes. A previous study showed that XBP1s can regulate the CCND1 promoter-driven reporter (CCND1). We tested the transcriptional activity of XBP1s by assessing CCND1 reporter luciferase activity in HEK293T cells overexpressing TRAF2 or XBP1s ( Figure 2B). The transcriptional activity of XBP1s was decreased by TRAF2 overexpression. When HEK293T cells were treated with TG, an ER stress inducer, the transcriptional activity of XBP1s was significantly reduced by the overexpression of TRAF2 ( Figure 2C). SCD is a desaturase that catalyzes the saturation of unsaturated fatty acids and is crucial for lipogenesis during ER stress. Previous studies have shown that XBP1s regulates SCD in mouse models [10]. Here, PA treatment enhanced SCD protein level, and TRAF2 overexpression suppressed PA-induced XBP1s and SCD expression in Lo2 cells ( Figure 2D). Furthermore, *trf-1* RNAi in transgenic worms, which expressed the ER stress reporter HSP4::GFP, induced hyperactivated ER stress signaling ( *xbp-1* dependent) upon dithiothreitol (DTT) treatment. Expression of the HSP4 promoter-driven reporter presented by GFP fluorescence (white) in *C* . *elegans* was increased by *trf-1* knockdown. Additionally, *xbp-1* knockdown inhibited the expression of the reporter, which was mediated by *trf-1* RNAi ( Figure 2E). Knockdown of *xbp-1* also decreased lipid accumulation in *C*. *elegans* ( Figure 2F). These findings indicate that TRAF2 can reduce the transcriptional activity of XBP1s and regulate lipid metabolism in mammals and worms and that this regulation is conserved between *H*. *sapiens* and *C*. *elegans*.

**Figure 2:**
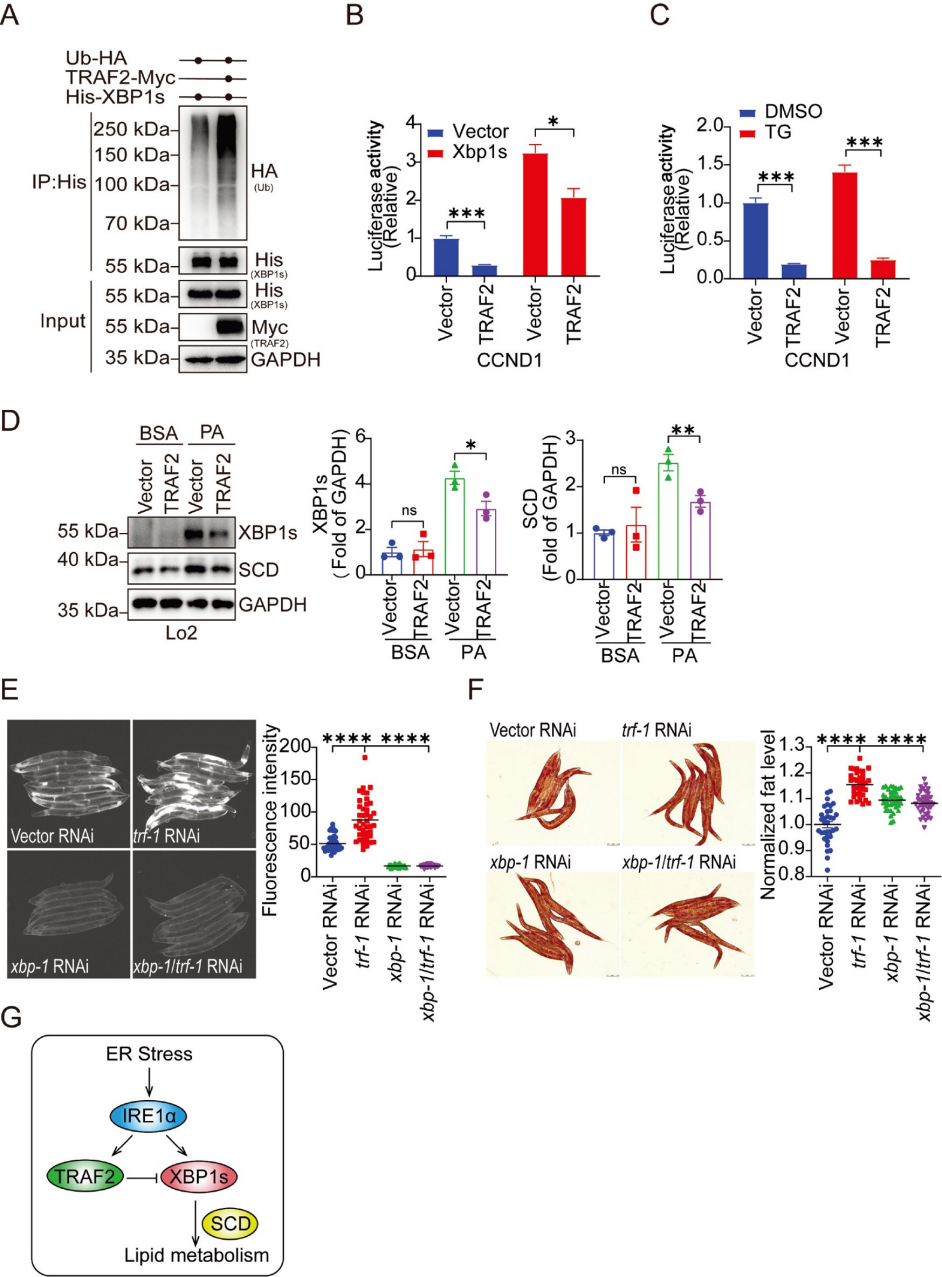
Figure 2 TRAF2 ubiquitinates and decreases the transcriptional activity of XBP1s (A) The HEK293T cell line was transfected with His-XBP1s together with or without TRAF2-Myc. Cell lysate was subjected to His bead precipitation and analyzed by western blot analysis. (B) HEK293T cells were transfected with CCND1 reporter together with or without XBP1s or TRAF2 for 24 h. Luciferase activity was detected using the Promage kit. (C) HEK293T cells were transfected with CCND1 reporter for 24 h and treated with TG (1 μM) for 6 h. The luciferase activity was detected using the Promage kit. (D) Lo2 cells were transfected with or without TRAF2 for 24 h and treated with PA (0.5 mM) for 24 h. The expressions of SCD and XBP1s were analyzed by western blot analysis (left) and quantified (right). (E) HSP4::GFP transgenic worms were fed with RNAi bacteria and treated with DTT (1 mM) for 3 h. The fluorescence was detected by microscopy. (F) Adult worms were fed with RNAi bacteria and stained with Oil Red O. (G) The schematic of TRAF2 and XBP1s signaling under ER stress. Data are presented as the mean±SEM from three independent experiments. *P<0.05, **P<0.01, ***P<0.001, and ****P<0.0001 (two-tailed unpaired Student’s t-test).

TRAF2 is regarded as the most significant adaptor in NF-κB signaling and can be activated by ER stress. Numerous studies have focused on the IRE1α/XBP1s pathway in the metabolism of nutrients, including glucose and lipids. It is well known that the regulation of ER stress signaling is complex. However, how TRAF2 and XBP1s, which are downstream of IRE1α, communicate with each other is unclear. Our results revealed a crosstalk between TRAF2 and XBP1s, the two arms of the IRE1α signaling pathway. The results of our study demonstrated that TRAF2 can ubiquitinate and inhibit the transcriptional activity of XBP1s. Although there is insufficient evidence indicating that TRAF2 is the direct E3 ligase of XBP1s, it may be valuable to study this further. The crosstalk between TRAF2 and XBP1s may regulate lipid metabolism in the liver. In conclusion, we revealed the IRE1α-TRAF2-XBP1s integrated signaling, which may be targeted to develop metabolism-related disease therapy.

## References

[1] Stefan N, Schick F, Birkenfeld AL, Häring HU, White MF (2023). The role of hepatokines in NAFLD. Cell Metab.

[2] Shao M, Shan B, Liu Y, Deng Y, Yan C, Wu Y, Mao T (2014). Hepatic IRE1α regulates fasting-induced metabolic adaptive programs through the XBP1s-PPARα axis signalling. Nat Commun.

[3] Martinez BA, Hoyle RG, Yeudall S, Granade ME, Harris TE, Castle JD, Leitinger N,
*et al*. Innate immune signaling in
*Drosophila* shifts anabolic lipid metabolism from triglyceride storage to phospholipid synthesis to support immune function.
*
PLoS Genet
* 2020, 16: e1009192. https://doi.org/10.1371/journal.pgen.1009192.

[4] Calfon M, Zeng H, Urano F, Till JH, Hubbard SR, Harding HP, Clark SG (2002). IRE1 couples endoplasmic reticulum load to secretory capacity by processing the XBP-1 mRNA. Nature.

[5] Chen ZJ (2005). Ubiquitin signalling in the NF-κB pathway. Nat Cell Biol.

[6] Hu P, Han Z, Couvillon AD, Kaufman RJ, Exton JH (2006). Autocrine tumor necrosis factor alpha links endoplasmic reticulum stress to the membrane death receptor pathway through IRE1α-mediated NF-κB activation and down-regulation of TRAF2 expression. Mol Cell Biol.

[7] Chen Z, Sheng L, Shen H, Zhao Y, Wang S, Brink R, Rui L (2012). Hepatic TRAF2 regulates glucose metabolism through enhancing glucagon responses. Diabetes.

[8] Wang Q, Zhou H, Bu Q, Wei S, Li L, Zhou J, Zhou S (2022). Role of XBP1 in regulating the progression of non-alcoholic steatohepatitis. J Hepatol.

[9] Lefebvre P, Lalloyer F, Baugé E, Pawlak M, Gheeraert C, Dehondt H, Vanhoutte J (2017). Interspecies NASH disease activity whole-genome profiling identifies a fibrogenic role of PPARα-regulated dermatopontin. JCI Insight.

[10] Lee AH, Scapa EF, Cohen DE, Glimcher LH (2008). Regulation of hepatic lipogenesis by the transcription factor XBP1. Science.

